# Sol–Gel Process Optimization of Transparent Silica Aerogels: Regulating Nanostructure to Enhance Optical Transmittance

**DOI:** 10.3390/nano16181137

**Published:** 2026-09-10

**Authors:** Guang Hu, Jiayang He, Rongjun Wu, Xiaoling Li, Yueyang Su, Boxuan Yang, Xu Wang, Jiadong Hu, Weiqiang Sun, Yihong Yan

**Affiliations:** 1 School of Nuclear Science and Technology, Xi’an Jiaotong University, Xi’an 710049, China; 2 Wuhan Second Ship Design and Research Institute, Wuhan 430064, China; 3 School of Human Settlements and Civil Engineering, Xi’an Jiaotong University, Xi’an 710049, China

**Keywords:** transparent nanoporous material, silica aerogel, sol–gel chemistry, optical transmittance

## Abstract

Silica aerogel, a prototypical transparent nanoporous material with ultralow density and a widely tunable refractive index, is employed as a Cherenkov radiator for precision gamma-ray diagnostics in inertial confinement fusion experiments. However, its optical transmittance is highly sensitive to sol–gel chemistry. Here, silica aerogels were prepared from tetraethyl orthosilicate (TEOS), ethanol, water, and aqueous ammonia, followed by aging, solvent exchange, and supercritical drying. The study examined how catalyst, ethanol, and water concentrations regulate nanostructure and optical transmittance. Increasing ammonia content accelerated gelation, produced smaller framework particles, and enlarged the pore size and pore volume. These changes increased the transmittance at 550 nm from 79.6% for C1 to 84.9% for C7. Increasing ethanol concentration diluted TEOS and its hydrolysis products, extended gelation, and narrowed the particle-size distribution. The average particle size decreased from 21.79 to 17.01 nm, while the transmittance at 550 nm increased from 57.4% for E1 to 65.2% for E5. Water produced a non-monotonic response: insufficient water limited TEOS hydrolysis, whereas excessive water promoted structural heterogeneity. Intermediate water content provided the most favorable optical response. These findings define a process–structure–transmittance relationship in which formulation-dependent nanoscale network formation governs scattering losses and optical clarity.

## 1. Introduction

Silica aerogels are three-dimensional nanoporous solids composed of interconnected silica clusters and air-filled pores. Their low density, high porosity, high specific surface area, and tunable refractive index make them useful in thermal insulation, optics, catalysis supports, and particle-radiation detector components. For optical applications, the value of silica aerogels lies not only in their low refractive index but also in the ability of the nanoporous network to transmit visible light with limited scattering. This makes transparent silica aerogels a process-sensitive material system in which synthesis chemistry directly determines final optical performance [1,2,3,4].

Sol–gel processing provides a flexible route to silica aerogels because hydrolysis and condensation of tetraethyl orthosilicate (TEOS) can be adjusted by solvent composition, water content, and catalyst concentration. These parameters affect the rate at which primary silica species form, aggregate, and connect into a percolated gel network. Subsequent aging, solvent exchange, and supercritical drying preserve the wet-gel network as an aerogel. The final optical transmittance is therefore expected to depend on a chain of coupled factors: precursor composition controls reaction kinetics; reaction kinetics control gelation time and cluster growth; cluster growth controls particle size, pore size, specific surface area, and pore volume; and those nanoscale structural parameters control scattering and transmission losses [2,5,6,7,8,9].

Silica aerogels have been considered as candidate Cherenkov radiators because weak optical-signal detection requires materials with high visible transmittance, and the aerogel refractive index can be selected through density control. This application therefore provides the motivation for the present work, but it is not the main subject of the manuscript. Instead, this study focuses on the preparation factors that improve optical transmittance in transparent silica aerogels. In particular, catalyst concentration, ethanol concentration, and water concentration are examined as formulation variables that regulate sol–gel network formation, adjust nanoscale pore structure, and consequently change optical transmittance [10,11].

This study systematically examines three formulation variables central to sol–gel synthesis: aqueous ammonia catalyst concentration, ethanol concentration, and water concentration. Catalyst concentration primarily modifies the alkaline hydrolysis-condensation environment; ethanol concentration mainly dilutes TEOS and its hydrolysis products; and water concentration controls the degree of TEOS hydrolysis while also affecting cluster uniformity when present in excess. By linking gelation behavior, scanning electron microscopy (SEM), particle-size distributions, nitrogen adsorption–desorption analysis, and transmittance at 550 nm, this study identifies the process–structure relationships that govern optical clarity in transparent silica aerogels [5,6,7,12].

## 2. Materials and Methods

### 2.1. Materials and Synthesis of Silica Aerogels

Silica aerogels were synthesized by a base-catalyzed sol–gel route using tetraethyl orthosilicate (TEOS; analytical grade; Chengdu Kelong Chemical Co., Ltd., Chengdu, China), absolute ethanol (EtOH; analytical grade; Chengdu Kelong Chemical Co., Ltd., Chengdu, China), deionized water (laboratory-produced), and aqueous ammonia (analytical grade; Chengdu Kelong Chemical Co., Ltd., Chengdu, China). In a typical synthesis, ethanol, water, and TEOS were mixed in a conical flask under magnetic stirring, after which aqueous ammonia was added as the catalyst. The sol was poured into glass molds and allowed to gel. Gelation time was defined as the interval from ammonia addition to the point at which the mold could be tilted by 30° without visible flow of the gel [2,5,8,9].

**Figure 1:**
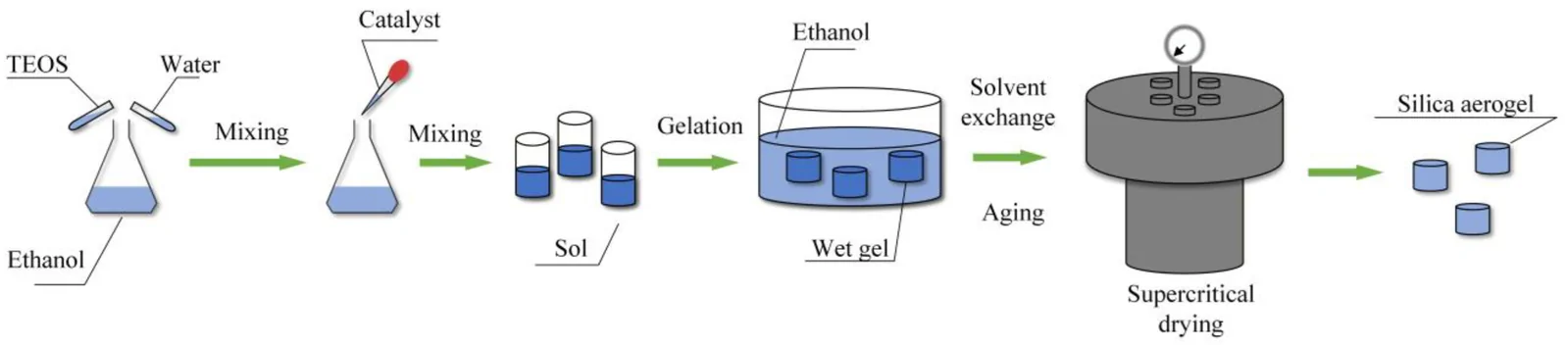
Schematic illustration of the silica aerogel preparation route, including sol–gel synthesis, aging, solvent exchange, and supercritical drying.

After gelation, the wet gels were placed in sealed ethanol-filled containers for aging and solvent exchange. Ethanol was replaced every other day to remove residual water and catalyst and to promote a more regular three-dimensional network. The gels were then dried by a supercritical drying process to obtain silica aerogels (Figure 1) [8,9].

### 2.2. Optical-Transmittance Measurement

**Figure 2:**
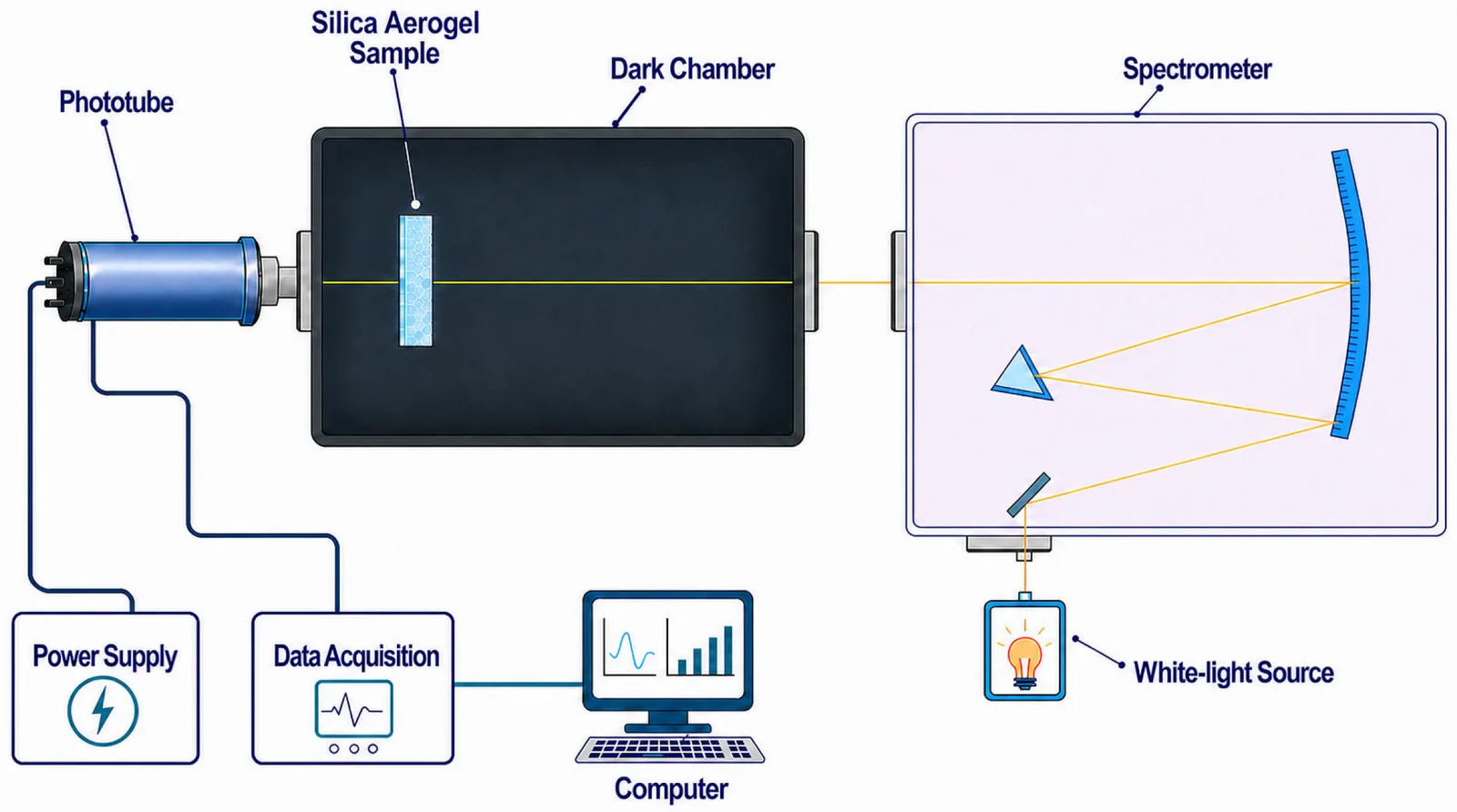
Experimental arrangement for optical-transmittance measurement using a white-light source, spectrometer, sample stage, phototube, and data acquisition system [13].

Optical transmittance was measured using the experimental arrangement shown in Figure 2, consisting of a white-light source, an Omni-300 spectrometer, a phototube, and a DCS300PA dual-channel data acquisition system (Zolix Instruments Co., Ltd., Beijing, China) in a dark chamber. The aerogel sample was mounted on a sample stage, and monochromatic light selected by the spectrometer was directed through the sample. The transmitted signal was recorded by the phototube and transferred to a computer through the data acquisition card. The wavelength interval was 1 nm, and the integration time was kept identical for the sample and source measurements at each wavelength. Repeated measurements of the white-light spectrum showed that the instability of the measurement system was below 2% [11].

The transmittance was calculated using

$$T\left(\lambda \right)=\frac{I_{signal}\left(\lambda \right)-I_{bk}\left(\lambda \right)}{I_{source}\left(\lambda \right)-I_{bk}\left(\lambda \right)}$$

where *I_signal_* is the signal measured through the aerogel, *I_bk_* is the dark background, and *I_source_* is the source intensity measured without the sample. For the concentration-optimization experiments, spectra were recorded over the 500–800 nm range, and transmittance at 550 nm was used as the comparative metric.

### 2.3. Microstructural and Surface Characterization

Microstructural observations and elemental mapping were carried out using an Ultra 55 field-emission scanning electron microscope (Carl Zeiss NTS GmbH, Oberkochen, Germany) equipped for energy-dispersive spectroscopy (EDS). Particle-size distributions were obtained from SEM images using Nano Measure software (version 1.2; Fudan University, Shanghai, China) by randomly selecting 500 aerogel framework particles. Fourier-transform infrared spectroscopy (FTIR) was performed using a Spectrum One spectrometer (PerkinElmer Inc., Waltham, MA, USA), and water contact angles were measured using a JC2000D3E contact-angle meter (Shanghai Zhongchen Digital Technic Apparatus Co., Ltd., Shanghai, China). Nitrogen adsorption–desorption isotherms were collected using an iPore-500 automatic physical adsorption analyzer (Physi-Chem Scientific Instruments (Beijing) Co., Ltd., Beijing, China). Specific surface areas were calculated by the Brunauer–Emmett–Teller (BET) method, and total pore volumes and average pore sizes were extracted from the adsorption data [14,15].

### 2.4. Experimental Design for Concentration Optimization

Three independent formulation series were designed to evaluate the effects of aqueous ammonia, ethanol, and water amounts. Within each series, only one component volume was varied, while the other components were held constant. Because the three series used different base formulations, their absolute properties were evaluated within each series rather than compared directly across series. The formulations and sample designations are summarized in Table 1.

## 3. Results

### 3.1. General Characterization of Silica Aerogels

Before optical-transmittance optimization, Samples 1–3 were characterized as independent reference specimens to establish the baseline properties of the prepared silica aerogels. They were not included in any of the C, E, or H optimization series. Samples 1, 2, and 3 had nominal bulk densities of 300, 119, and 33 mg·cm^−3^, respectively, determined by the conventional mass-to-volume method, ρ = m/V, where m is the dry mass measured with an analytical balance, and V is the geometric volume calculated from the measured macroscopic dimensions. Their molar formulations (TEOS:H_2_O:EtOH:NH_4_OH) were 1:0.3:1:1.1 × 10^−3^, 1:1.6:5:1.1 × 10^−3^, and 1:1.2:15:1.1 × 10^−3^, respectively.

**Figure 3:**
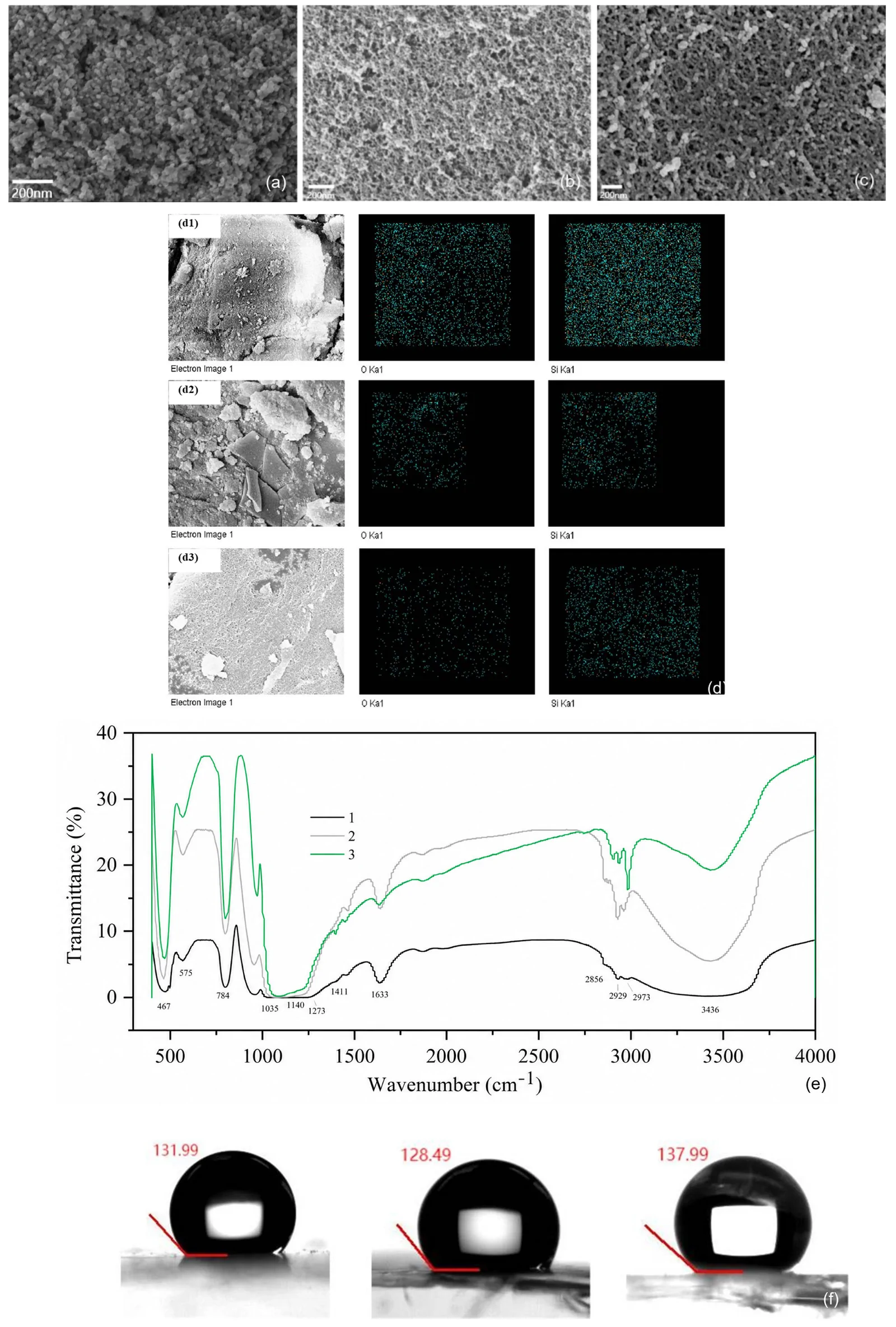
Preliminary characterization of three independent reference aerogels: (**a**–**c**) SEM images of Samples 1–3; (**d1**–**d3**) EDS elemental mapping of Samples 1–3, arranged from top to bottom; (**e**) FTIR spectra, with curves 1, 2, and 3 corresponding to Samples 1, 2, and 3; and (**f**) water-contact-angle results for Samples 1–3.

These reference specimens were used to verify the nanoporous framework morphology and elemental composition of the prepared aerogels before evaluating the effects of aqueous ammonia, ethanol, and water. SEM images (Figure 3a–c) showed that the framework particle size decreased with decreasing bulk density, consistent with the reduction in solid skeletal fraction. This structural variation is relevant to optical transmission because both framework particle size and pore size influence light scattering. EDS mapping (Figure 3d1–d3) further showed uniform distributions of Si and O throughout the aerogel framework, with no obvious elemental aggregation or detectable extraneous elements in the observed regions [11,12].

The FTIR curves labeled 1, 2, and 3 in Figure 3e correspond to Samples 1, 2, and 3 (300, 119, and 33 mg·cm^−3^), respectively. The spectra showed bands attributable to hydroxyl, organic, and Si–O-related vibrations, including broad features near 3436 and 1411 cm^−1^, a band near 1035 cm^−1^, and lower-wavenumber bands near 467 and 575 cm^−1^. The intensity of the spectral bands decreased with decreasing aerogel density, consistent with lower solid framework content. Water contact-angle measurements of Samples 1–3 (Figure 3f) indicated hydrophobic surfaces at all three density levels, attributed to residual hydrophobic groups on the silica framework.

### 3.2. Effect of Catalyst Concentration

**Figure 4:**
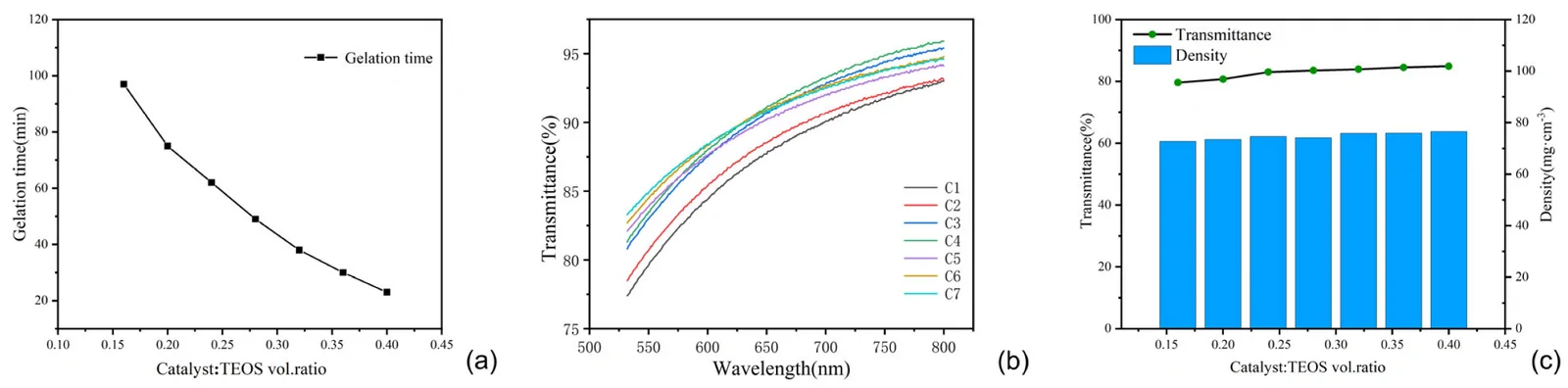
Effect of the catalyst/TEOS volume ratio on the catalyst series: (**a**) gelation time; (**b**) optical-transmittance spectra; and (**c**) variations in T_550_ and bulk density.

Increasing catalyst concentration shortened the gelation time (Figure 4a). Under alkaline conditions, ammonia promotes deprotonation of silanol groups to form silanolate species (Si–O^−^). These strong nucleophiles promote Si–O–Si bond formation through condensation. Therefore, the shorter gelation time observed at higher aqueous ammonia concentration is attributed primarily to enhanced condensation kinetics rather than to uniform acceleration of both hydrolysis and condensation. The higher concentration of reactive silanolate species facilitates network-forming reactions and gel-network formation, whereas fewer such species at low catalyst concentration delays gelation [5,6,7].

The optical-transmittance spectra (Figure 4b) and the 550 nm summary (Figure 4c) showed a positive relationship between catalyst concentration and visible transmittance. At 550 nm, sample C1 exhibited a transmittance of approximately 79.6%, whereas the 10 mm-thick C7 sample reached approximately 84.9%. Thus, within the catalyst range tested here, increasing ammonia concentration improved optical transmittance while also reducing gelation time. This combination is practically useful because it improves both material transparency and preparation efficiency.

**Figure 5:**
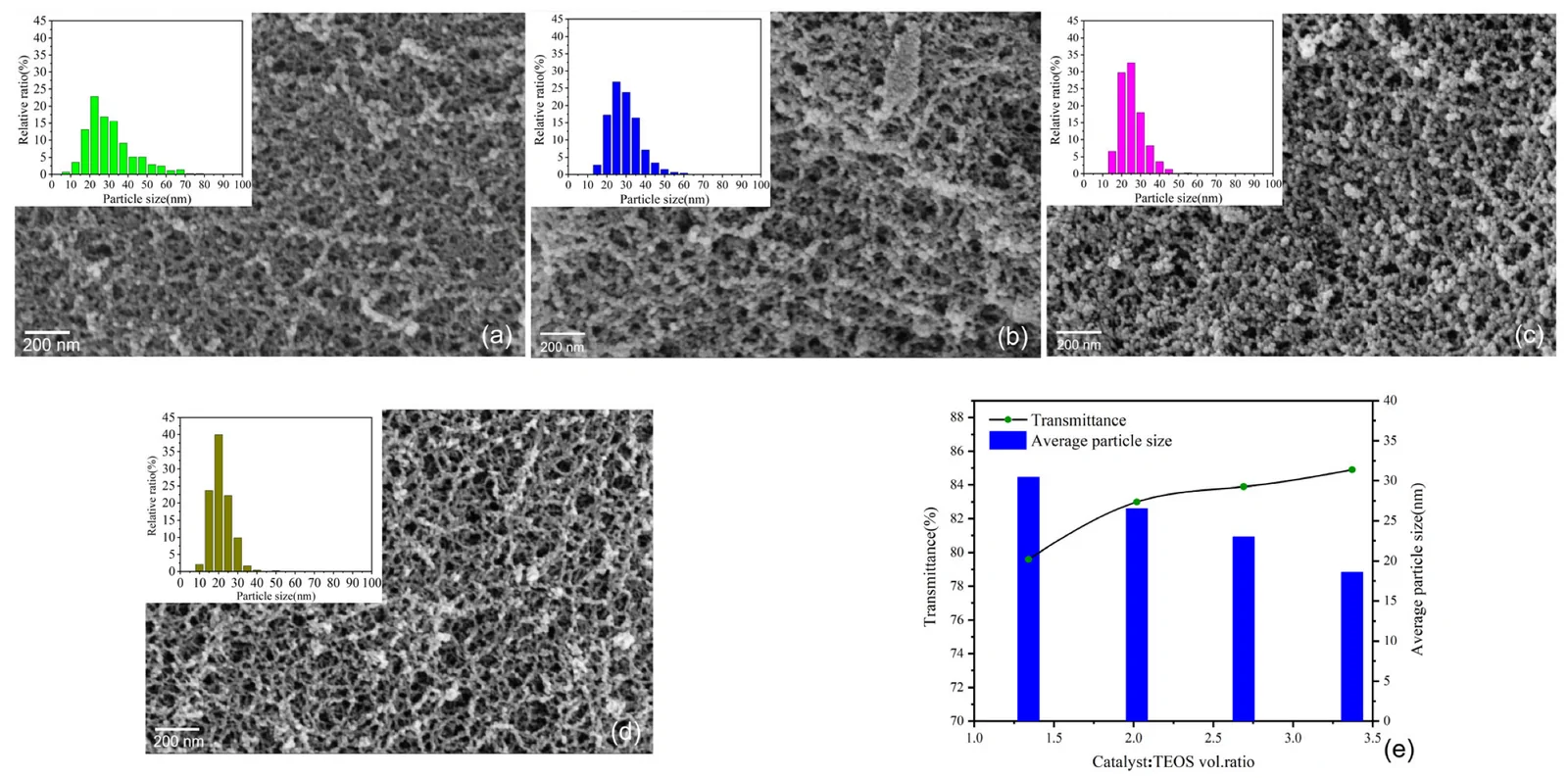
SEM micrographs and particle-size/transmittance response of catalyst-series aerogels: (**a**) C1; (**b**) C3; (**c**) C5; (**d**) C7; and (**e**) average particle size and T_550_.

The SEM micrographs for C1, C3, C5, and C7 (Figure 5a–d), together with the particle-size/transmittance summary (Figure 5e), linked the transmittance improvement to the refinement of the aerogel network. At low catalyst concentration, C1 showed relatively large framework particles, low apparent porosity, and an inhomogeneous network. This morphology is consistent with slow polymerization, which allows small TEOS-derived clusters to deposit onto a preformed gel framework, increases bulk polymerization, and enlarges skeletal features. As catalyst concentration increased, the framework particles became smaller, the network became more pronounced, and the pore structure became more open. For C7, nearly 40% of the particles were distributed near 20 nm in the particle-size analysis.

**Figure 6:**
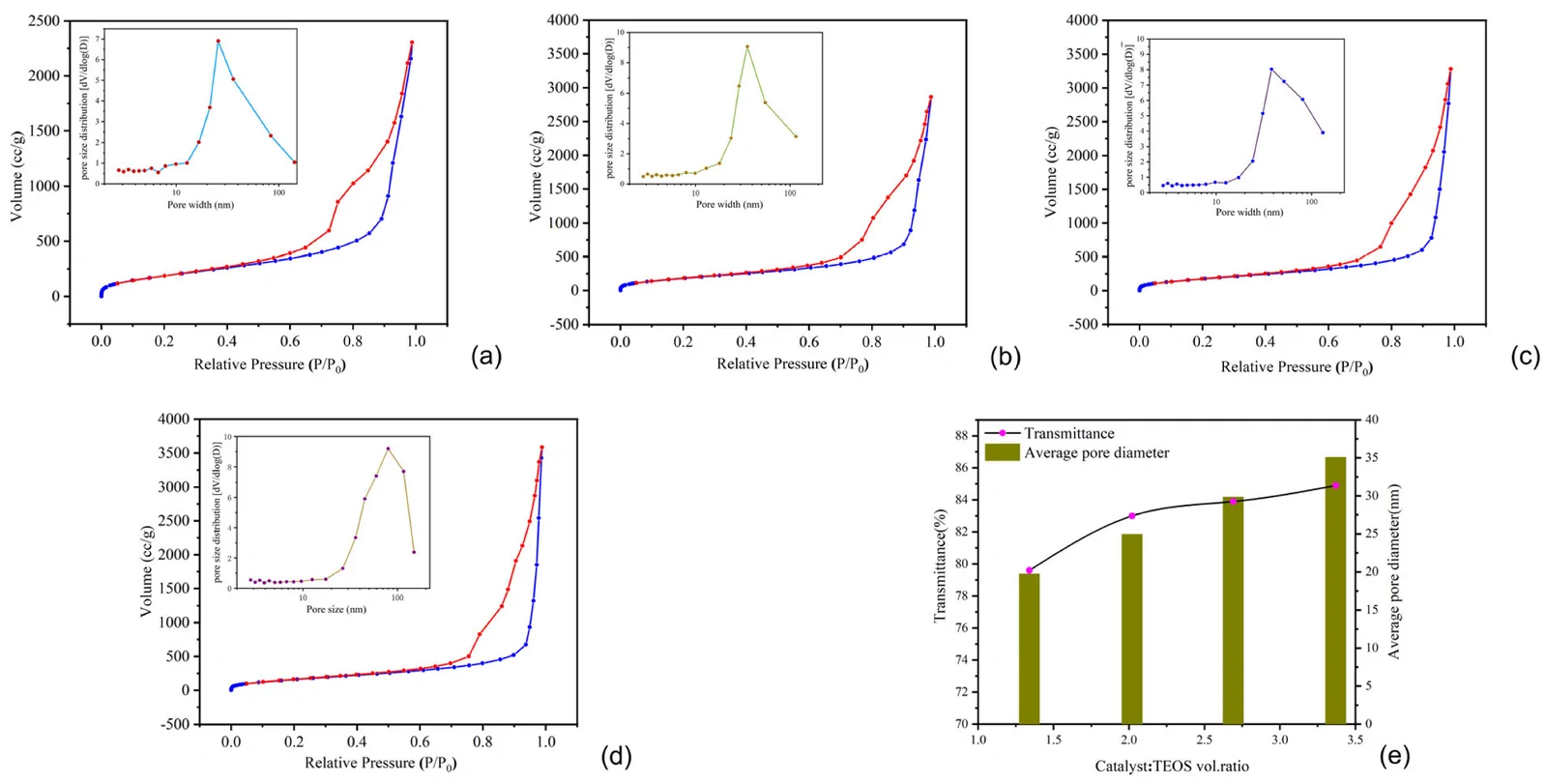
Nitrogen adsorption–desorption isotherms, pore-size distributions and pore-structure/transmittance response of catalyst-series aerogels: (**a**) C1; (**b**) C3; (**c**) C5; (**d**) C7; and (**e**) average pore size and T_550_. In panels (**a**–**d**), the blue lower branch denotes adsorption, whereas the red upper branch denotes desorption. In each inset, the line represents the pore-size-distribution curve and the symbols represent measured data points.

Nitrogen adsorption–desorption data further supported the microstructural interpretation. The catalyst-series isotherms and pore-size distributions for C1, C3, C5, and C7 (Figure 6a–d), together with the pore-size/transmittance summary (Figure 6e), exhibited hysteresis loops at relative pressures of P/P_0_ = 0.6–0.9, characteristic of type IV isotherms and confirming the mesoporous structure [15].

The quantitative pore-structure parameters are summarized in Table 2. As catalyst concentration increased from C1 to C7, the BET surface area decreased from 728.7 to 632.7 m^2^·g^−1^, while total pore volume increased from 3.560 to 5.545 cm^3^·g^−1^ and average pore size increased from 19.74 to 35.06 nm. These changes indicate that higher catalyst concentration produced a more open network with larger pores and reduced specific surface area.

### 3.3. Effect of Ethanol Concentration

**Figure 7:**
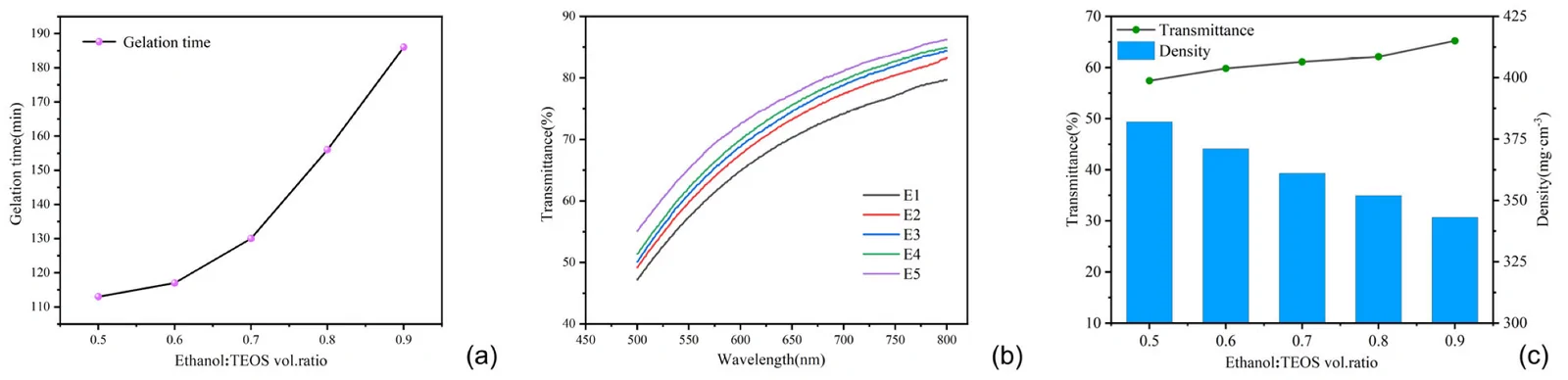
Effect of ethanol concentration on the ethanol series: (**a**) gelation time; (**b**) optical-transmittance spectra; and (**c**) variations in T_550_ and bulk density.

Ethanol concentration had the opposite effect on gelation time compared with catalyst concentration (Figure 7a). When TEOS, water, and ammonia content were fixed, increasing ethanol diluted the reacting species and reduced the probability of contact between TEOS-derived species and water molecules. As a result, TEOS hydrolysis and condensation slowed, and gelation time increased [6,7].

The ethanol-series transmittance spectra increased with wavelength in the visible range (Figure 7b), and the 550 nm summary showed improved transmittance as ethanol concentration increased (Figure 7c). At 550 nm, E1 showed a transmittance of approximately 57.4%, whereas E5 reached approximately 65.2%. The measured aerogel density also decreased as ethanol concentration increased because the relative amount of TEOS-derived skeletal material decreased. Lower density and more open pores can reduce optical obstruction, but the SEM results show that the more important optical factor is the improved uniformity of the framework.

**Figure 8:**
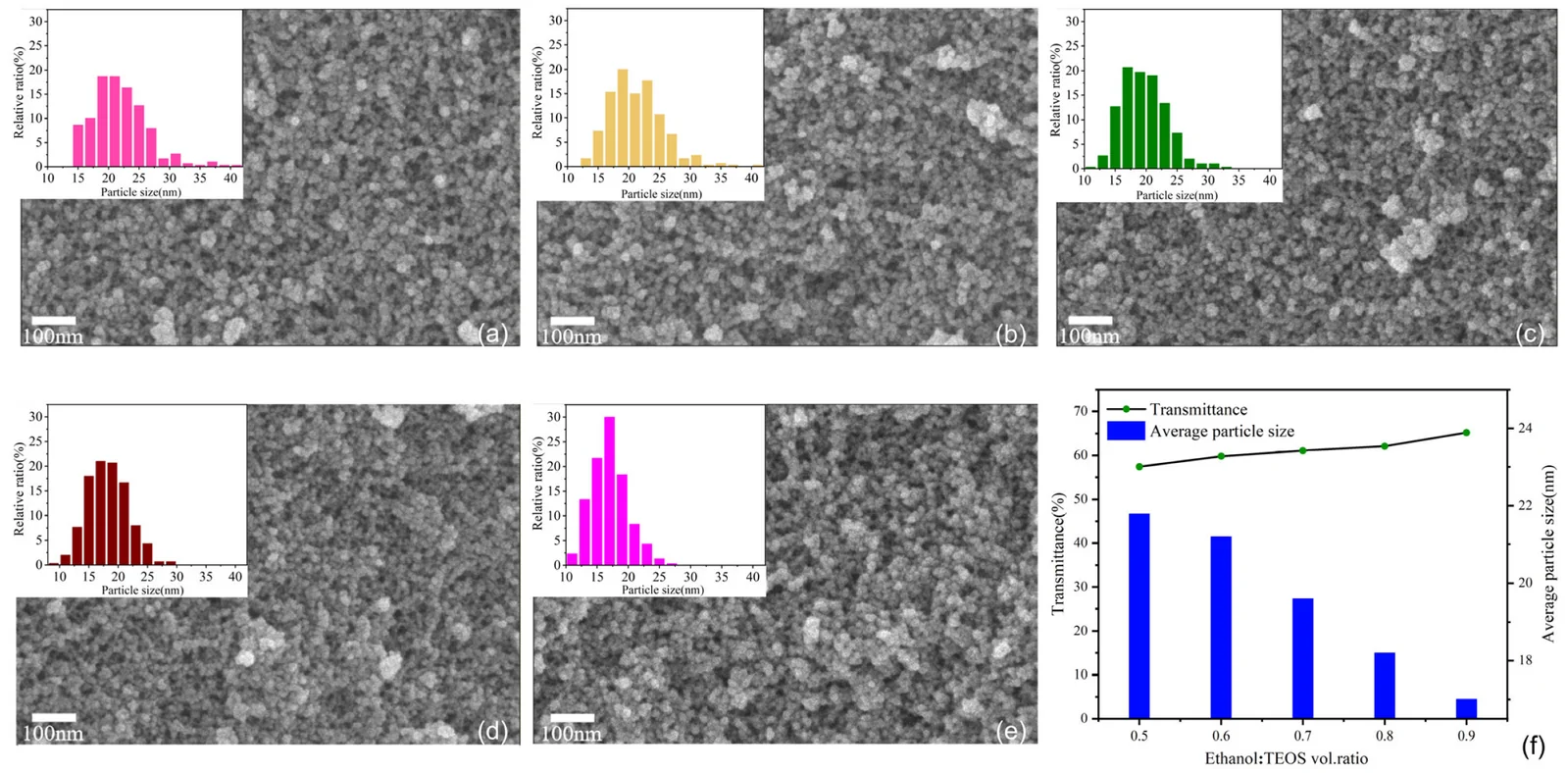
SEM micrographs and particle-size/transmittance response of ethanol-series aerogels: (**a**) E1; (**b**) E2; (**c**) E3; (**d**) E4; (**e**) E5; and (**f**) average particle size and T_550_.

The SEM micrographs for E1–E5 (Figure 8a–e) and the particle-size/transmittance summary (Figure 8f) revealed that E1 contained relatively large framework particles and a broad particle-size distribution, consistent with rapid local aggregation at higher TEOS concentration. Such nonuniform skeletal particles generate nonuniform pore structures, which enhance scattering and lower transmittance. With increasing ethanol concentration, the framework became more homogeneous, and the particle-size distribution shifted toward smaller sizes. The average particle size decreased from 21.79 nm for E1 to 17.01 nm for E5. This refinement and homogenization reduced visible-light scattering and explains the increase in 550 nm transmittance.

**Figure 9:**
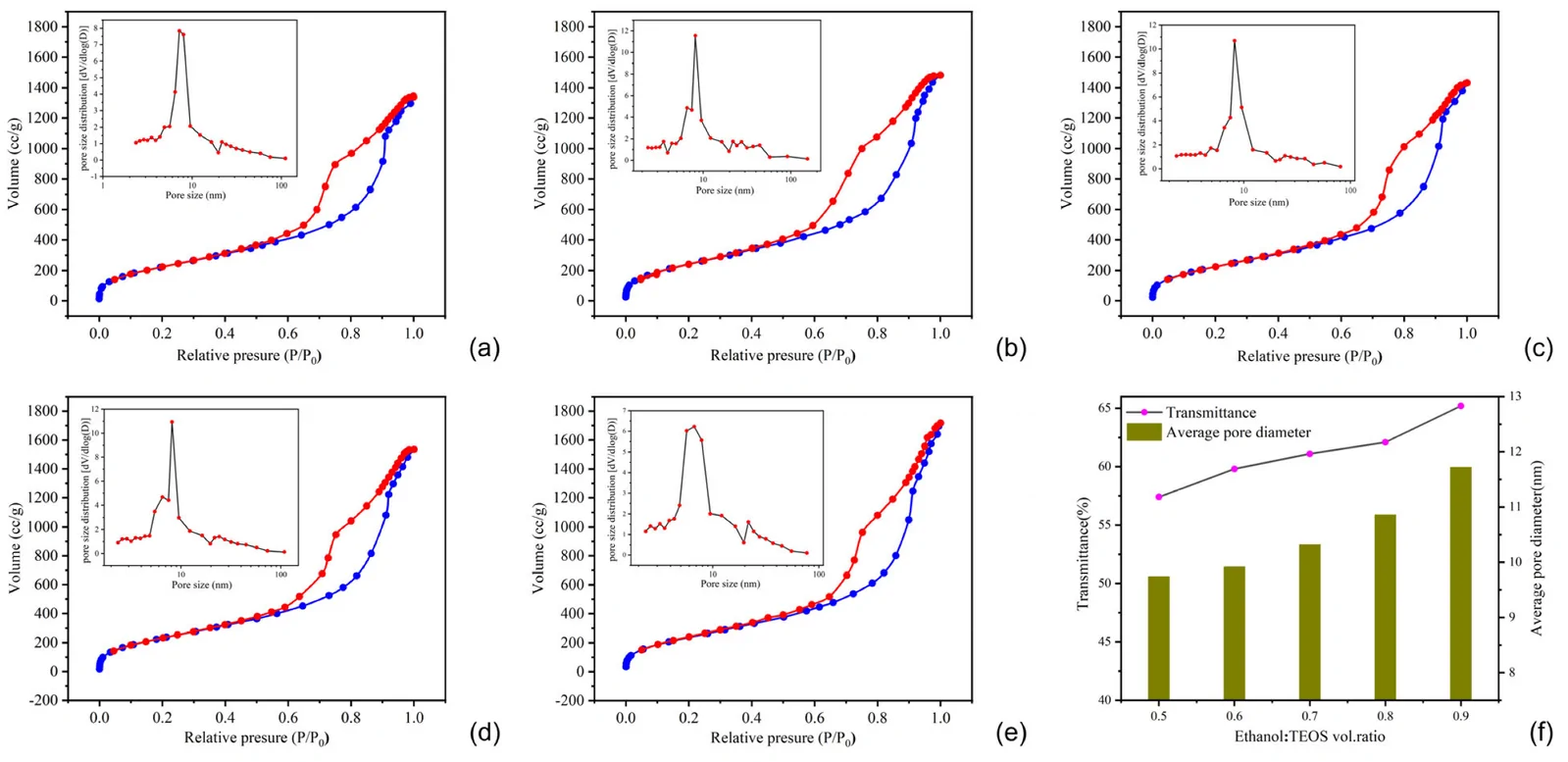
Nitrogen adsorption–desorption isotherms, pore-size distributions and pore-structure/transmittance response of ethanol-series aerogels: (**a**) E1; (**b**) E2; (**c**) E3; (**d**) E4; (**e**) E5; and (**f**) average pore size and T_550_. In panels (**a**–**e**), the blue lower branch denotes adsorption, whereas the red upper branch denotes desorption. In each inset, the black line represents the pore-size-distribution curve and the red symbols represent measured data points.

Nitrogen adsorption–desorption isotherms and pore-size distributions for E1–E5 (Figure 9a–e), together with the pore-size/transmittance summary (Figure 9f), were also described as type IV isotherms [15].

The corresponding pore-structure parameters are summarized in Table 3. The average pore size increased from 9.74 nm for E1 to 11.72 nm for E5, while total pore volume increased from 2.07 to 2.66 cm^3^·g^−1^. The BET surface area increased from E1 to E5 overall, although the intermediate samples did not follow a strictly monotonic sequence. This behavior is consistent with dilution of the reaction system: ethanol occupies space between developing gel clusters, disperses the skeleton, and leaves additional pore volume after drying.

### 3.4. Effect of Water Concentration

**Figure 10:**
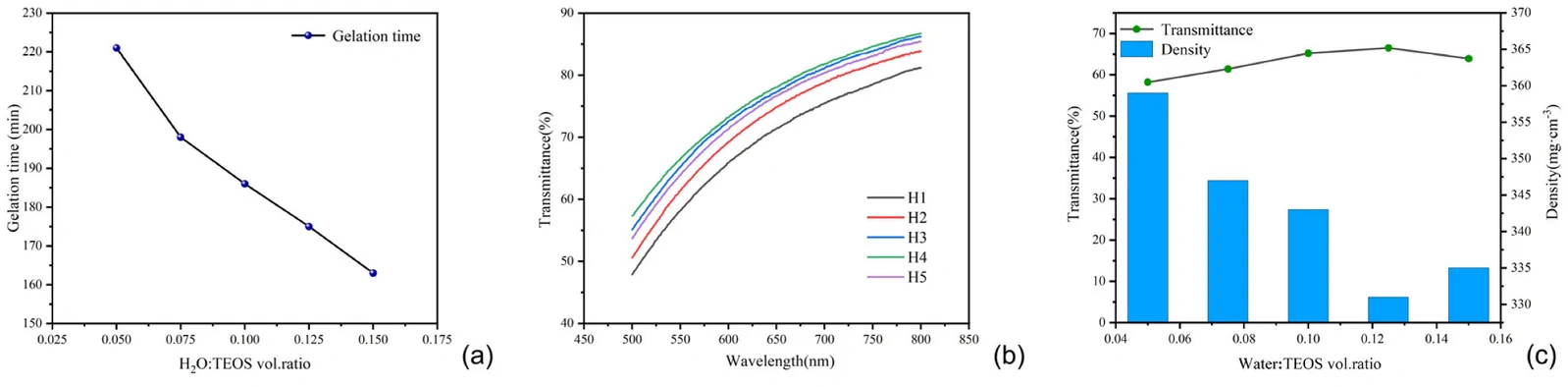
Effect of the water/TEOS volume ratio on the water series: (**a**) gelation time; (**b**) optical transmittance spectra; and (**c**) variations in T_550_ and bulk density.

Water concentration produced a non-monotonic optical response (Figure 10a–c). Gelation time decreased as the water-to-TEOS amount increased because higher water content accelerated TEOS hydrolysis and increased its extent, producing smaller silicate species that could participate in condensation more readily. However, unlike the catalyst and ethanol series, optical transmittance did not increase across the full water range [5,6,7].

At low water content, TEOS hydrolysis was incomplete, and partially hydrolyzed TEOS molecules participated directly in condensation to form larger framework particles. This led to lower transmittance: H1 had a 550 nm transmittance of approximately 58.2%. As water content increased, hydrolysis became more complete, the framework particle size decreased, the pore structure improved, and transmittance increased. At still higher water contents, the decrease in transmittance may be associated with repulsive interactions and compositional inhomogeneity in the solution, which generated molecular clusters of different sizes and led to nonuniform skeletal particles. H5 transmittance decreased to approximately 63.9% at 550 nm.

**Figure 11:**
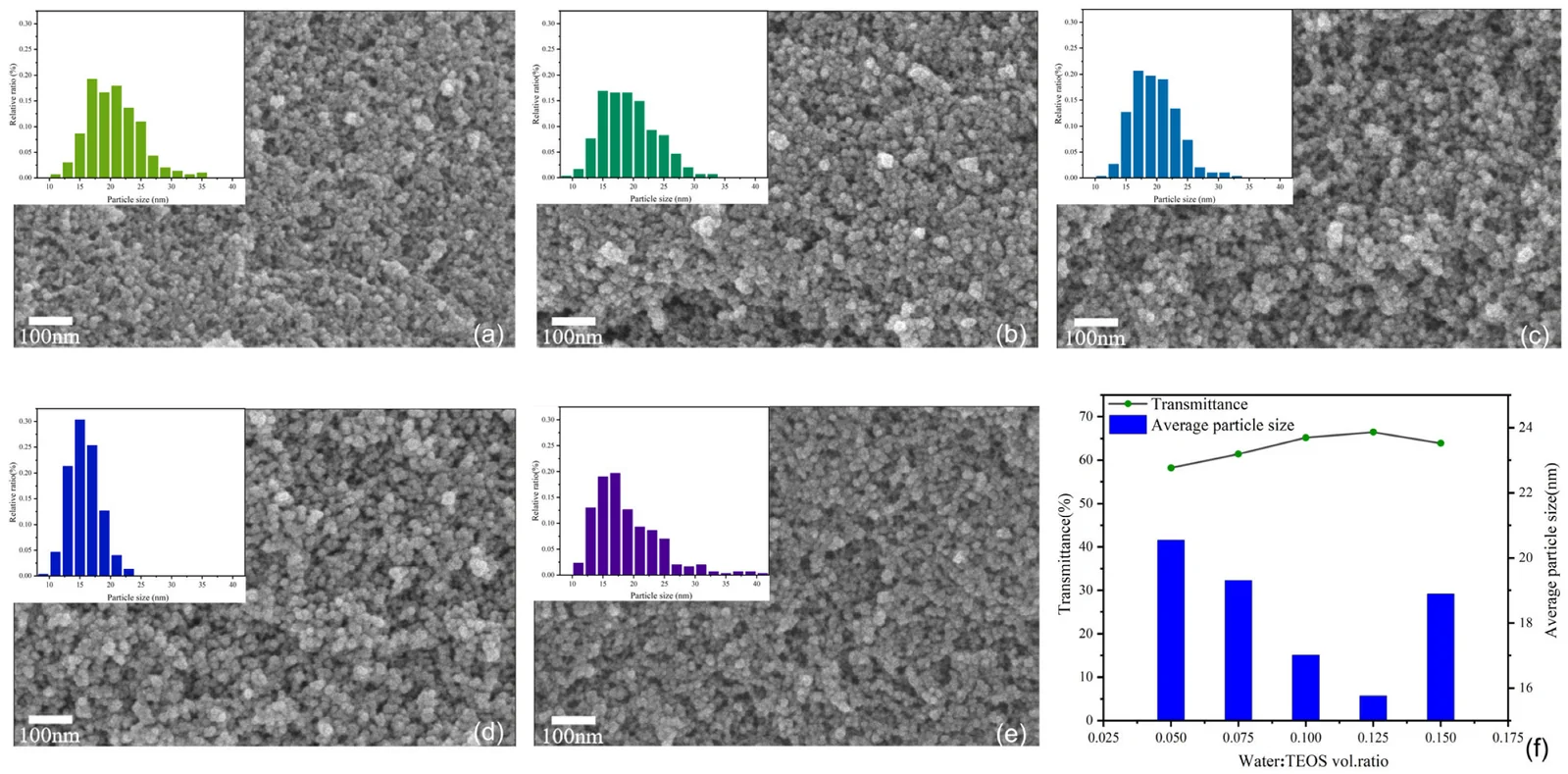
SEM micrographs and particle-size/transmittance response of water-series aerogels: (**a**) H1; (**b**) H2; (**c**) H3; (**d**) H4; (**e**) H5; and (**f**) average particle size and T_550_.

The SEM micrographs for H1–H5 (Figure 11a–e) and the particle-size/transmittance summary (Figure 11f) supported this mechanism. As water concentration increased from a deficient level, the framework particle size first decreased, and visible-light scattering was reduced. At excessive water content, the framework became less uniform because clusters with different sizes formed in the sol before gelation. Consistent with this interpretation, the particle-size analysis showed a decrease and then an increase in average particle size, which paralleled the increase and subsequent decrease in transmittance.

**Figure 12:**
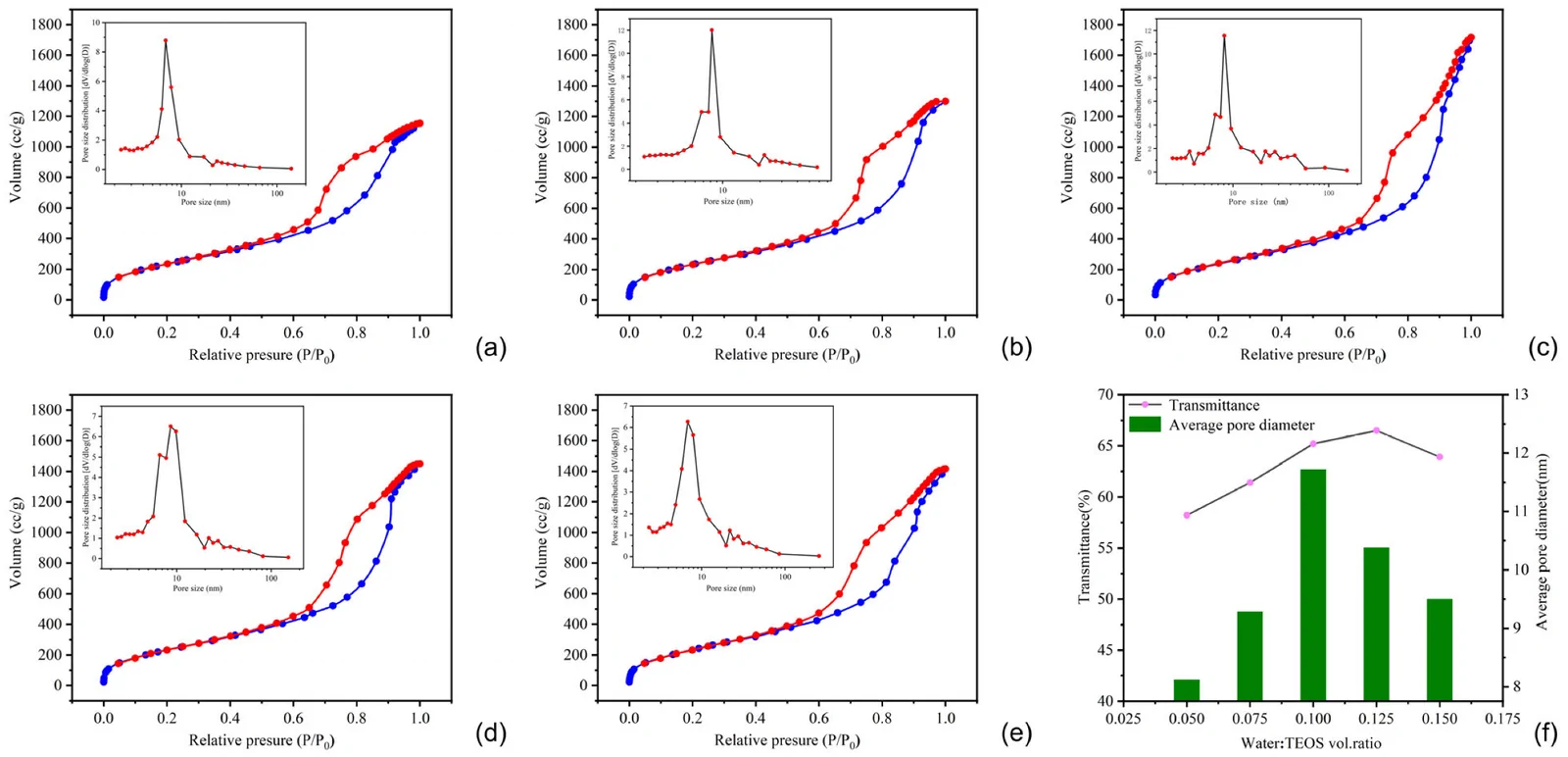
Nitrogen adsorption–desorption isotherms, pore-size distributions and pore-structure/transmittance response of water-series aerogels: (**a**) H1; (**b**) H2; (**c**) H3; (**d**) H4; (**e**) H5; and (**f**) average pore size and T_550_. In panels (**a**–**e**), the blue lower branch denotes adsorption, whereas the red upper branch denotes desorption. In each inset, the black line represents the pore-size-distribution curve and the red symbols represent measured data points.

The water-series nitrogen adsorption–desorption curves and pore-size distributions for H1–H5 (Figure 12a–e), together with the pore-size/transmittance summary (Figure 12f), were classified as type IV isotherms. Table 4 summarizes the BET surface area, total pore volume, and average pore size of the water-series samples. The BET surface area varied non-monotonically between 863.4 and 922.4 m^2^·g^−1^. Total pore volume increased from 1.78 cm^3^·g^−1^ for H1 to 2.66 cm^3^·g^−1^ for H3, then decreased to 2.19 cm^3^·g^−1^ for H5. The average pore size followed a similar trend, increasing from 8.12 nm for H1 to 11.72 nm for H3 before decreasing to 9.504 nm for H5. Figure 12f shows that T_550_ increased from 58.2% for H1 to 66.5% for H4 and then decreased to 63.9% for H5. Together, these non-monotonic trends indicate an optimum water-content window rather than a response governed by a single pore-structure parameter [15].

## 4. Discussion

### 4.1. Sol–Gel Composition Controls Network Formation

The three concentration variables affect different stages of the TEOS sol–gel pathway and therefore should not be treated as equivalent tuning parameters. Aqueous ammonia mainly changes the alkaline catalytic environment. Higher ammonia concentration increases the formation of silanolate species (Si–O^−^), which act as strong nucleophiles and promote Si–O–Si bond formation through condensation. Consequently, gelation is shortened mainly because condensation kinetics are enhanced, rather than because hydrolysis and condensation are uniformly accelerated. Within the catalyst range examined here, the enhanced condensation did not lead to visible macroscopic inhomogeneity; instead, it produced a finer framework and a more open network. Ethanol acts mainly as a diluent and co-solvent. By reducing the effective concentration of TEOS-derived species and water in the reacting medium, it lowers the probability of local overgrowth and large-cluster formation, producing a more homogeneous skeleton but increasing gelation time. Water directly determines the degree of TEOS hydrolysis. Its effect exhibits an optimum window rather than a monotonic trend: too little water leaves hydrolysis incomplete, whereas excessive water may disturb the balance between hydrolysis and condensation and promote structural heterogeneity before gelation [5,6,7,8,9].

### 4.2. Structural Origin of the Optical-Transmittance Trend

The changes in optical transmittance are better understood as the combined result of framework particle coarsening, structural heterogeneity, and refractive-index fluctuation rather than as the consequence of a single pore-size parameter. In transparent aerogels, visible-light loss is dominated by scattering from nanoscale density fluctuations in the silica–air network. Larger or more unevenly distributed framework particles create stronger local refractive-index contrast and broaden the characteristic scattering length scale, thereby reducing transmittance. Conversely, a finer and more uniformly connected framework suppresses spatial fluctuations in refractive index and improves optical clarity. This explains why the catalyst and ethanol series show higher T_550_ when their particle-size distributions become smaller and narrower [11,12].

The role of pore structure should therefore be interpreted with caution. Larger average pores do not automatically improve transparency. In the catalyst series, the increase in average pore size occurred together with a reduction in framework particle size and an increase in network openness; the transmittance improvement is therefore more plausibly governed by the reduced skeletal coarseness and improved structural uniformity than by pore enlargement alone. Similarly, in the ethanol series, dilution improved transmittance primarily by narrowing the particle-size distribution and reducing local aggregation. In the water series, the same mechanism appears to operate only up to an optimum composition window. Beyond that window, the decrease in transmittance is more reasonably attributed to increased network heterogeneity and renewed particle-size broadening. Because the proposed cluster formation under excess water was inferred from ex situ morphology and adsorption data rather than directly observed during sol formation, it should be regarded as a plausible mechanism that requires further confirmation by in situ or solution-state characterization [12,16].

### 4.3. Comparative Assessment of the Three Formulation Variables

The absolute T_550_ values of the catalyst, ethanol, and water series should not be ranked directly because their base formulations and density ranges are not identical. A more useful comparison is the role each variable plays within its own formulation window. Catalyst concentration is the most efficient parameter for shortening gelation time while improving transparency in the tested medium-density formulation. Ethanol concentration improves framework uniformity but introduces a preparation-time penalty. Water concentration is the most sensitive variable because both insufficient and excessive water contents are detrimental to optical clarity. The practical optimization strategy is therefore not to maximize one component independently, but to balance reaction rate, skeleton uniformity, density, and pore development [10,12]. The distinct roles, structural effects, optical responses, and processing implications of the three formulation variables are summarized in Table 5.

### 4.4. Comparison with Recent Transparent Silica Aerogel Studies

Recent studies on transparent silica aerogels generally show that optical performance is controlled by the suppression of scattering centers rather than by porosity alone. Wang et al. summarized that transparent thermal-insulation aerogels require simultaneous control of network size, pore uniformity, and haze, which is consistent with the present process-structure interpretation [16]. The best sample in this work, C7, reached a T_550_ of 84.9% for a 10 mm-thick aerogel. This value is comparable to or higher than many recent transparent silica aerogel reports when the comparison is made at a similar visible wavelength. For example, An et al. reported a broad-spectrum transparency of about 70% from 400 to 800 nm for transparent silica aerogel materials prepared with hydrolysis control and silylation-assisted ambient-pressure drying [17], and Marszewski et al. reported nanoparticle-based silica aerogel slabs with visible transmittance exceeding 75% for 5 mm-thick monoliths synthesized under near-ambient conditions [18]. Abebe et al. reported hybrid silica aerogels with visible-region transparency exceeding 80% and about 80% transmittance at 550 nm for the optimized BT2 composition, together with very low thermal conductivity [19]. These values indicate that the T_550_ obtained here is within a competitive range for transparent monolithic silica aerogels [16,20,21,22].

Some reported values equal or exceed the present result, but direct comparison is limited by differences in measurement conditions and synthesis routes. Zhang et al. obtained a solar-weighted transmittance of 83% after catalyst and TMOS-precursor refinement [23]; this metric integrates a broad solar spectrum and is therefore not identical to the single-wavelength T_550_ used here. Shi et al. reported a transmittance of 70% at 550 nm for a 0.25 g·cm^−3^ sample and a much higher value of 97.78% at 800 nm [24]. The latter should not be compared directly with T_550_ because Rayleigh-type scattering decreases strongly at longer wavelengths, so 800 nm transmittance is naturally less penalized by nanoscale scattering than 550 nm transmittance. Xia et al. increased the transparency ratio from 71% to 88% at 550 nm by using batch addition of TMOS to refine the microstructure [25]. That value is slightly higher than the C7 value in this work, but it was achieved through a different precursor-feeding strategy designed specifically to control cluster growth at constant composition and density. Therefore, the present contribution should not be positioned as a record optical-transmittance study. Its main value is the systematic separation of aqueous ammonia, ethanol, and water concentration effects in a TEOS-based sol–gel route, clarifying how each variable affects gelation, nanoscale framework formation, and visible-light scattering. A side-by-side comparison of the present work with these representative transparent silica aerogel studies is provided in Table 6.

## 5. Conclusions

Transparent silica aerogels were prepared by an ammonia-catalyzed TEOS sol–gel route to determine how catalyst, ethanol, and water concentrations regulate nanostructure and optical transmittance. Increasing catalyst concentration shortened gelation, refined the framework particles, increased pore openness, and raised the 550 nm transmittance from 79.6% for C1 to 84.9% for C7. Increasing ethanol concentration extended gelation but produced a finer and more uniform framework. The average particle size decreased from 21.79 to 17.01 nm, while the 550 nm transmittance increased from 57.4% for E1 to 65.2% for E5. Water showed an optimum composition window. Insufficient water limited TEOS hydrolysis, whereas excessive water promoted structural heterogeneity and reduced transmittance. Overall, optical transmittance was governed by the combined control of framework particle coarsening, network heterogeneity, and refractive-index fluctuations rather than by a single structural parameter. These results identify complementary formulation-based strategies for improving aerogel transparency and show that optical transmittance is sensitive to all three variables. The three-factor analysis provides an experimental basis and practical guidance for preparing highly transparent silica aerogels through coordinated control of reaction kinetics and structural uniformity. This process-level understanding may support high-transparency aerogels for advanced optical systems and aerogel-based components relevant to inertial confinement fusion.

## Figures and Tables

**Table 1:** Experimental formulations for the catalyst-, ethanol-, and water-optimization series.

Series (Samples)	TEOS (mL)	H_2_O (mL)	EtOH (mL)	Aqueous Ammonia (mL)
Catalyst series (C1–C7)	1	1.86	9	0.16–0.40 (Δ = 0.04)
Ethanol series (E1–E5)	10	1	5–9 (Δ = 1)	0.032
Water series (H1–H5)	10	0.50–1.50 (Δ = 0.25)	9	0.032

Note: Δ denotes the increment in the volume of the varied component between two consecutive samples within each series. Sample numbers increase with the amount of the varied component. Thus, C1–C7 correspond to aqueous ammonia volumes of 0.16–0.40 mL with Δ = 0.04 mL, E1–E5 correspond to ethanol volumes of 5–9 mL with Δ = 1 mL, and H1–H5 correspond to water volumes of 0.50–1.50 mL with Δ = 0.25 mL, respectively.

**Table 2:** BET surface area, total pore volume, and average pore size of catalyst-series samples.

Sample	BET Surface Area (m^2^·g^−1^)	Total Pore Volume (cm^3^·g^−1^)	Average Pore Size (nm)
C1	728.7	3.560	19.74
C3	710.1	4.431	24.96
C5	680.4	5.075	29.84
C7	632.7	5.545	35.06

**Table 3:** BET surface area, total pore volume, and average pore size of ethanol-series samples.

Sample	BET Surface Area (m^2^·g^−1^)	Total Pore Volume (cm^3^·g^−1^)	Average Pore Size (nm)
E1	848.9	2.07	9.74
E2	924.2	2.29	9.92
E3	858.4	2.21	10.32
E4	874.2	2.37	10.86
E5	906.7	2.66	11.72

**Table 4:** BET surface area, total pore volume, and average pore size of water-series samples.

Sample	BET Surface Area (m^2^·g^−1^)	Total Pore Volume (cm^3^·g^−1^)	Average Pore Size (nm)
H1	880.0	1.78	8.12
H2	865.42	2.01	9.28
H3	906.7	2.66	11.72
H4	863.4	2.24	10.386
H5	922.4	2.19	9.504

**Table 5:** Comparative effects of the three formulation variables on transparent silica aerogels.

Variable	Main Role in Sol–Gel Process	Dominant Structural Effect	Effect on T_550_ Within Tested Range	Main Processing Implication
Aqueous ammonia	Promotes silanol deprotonation and accelerates condensation	Smaller framework particles; more open pore network	Monotonic increase from 79.6% to 84.9%	Useful for both faster gelation and higher transparency; excessive catalyst beyond the tested range should be checked for inhomogeneity
Ethanol	Dilutes TEOS-derived species and reduces local aggregation	Narrower particle-size distribution; reduced average particle size	Monotonic increase from 57.4% to 65.2%	Improves optical uniformity but lengthens gelation time and changes density
Water	Controls the degree of TEOS hydrolysis	Optimum-window behavior; deficiency or excess increases heterogeneity	Increases to a maximum and then decreases	Most sensitive variable; requires a bounded composition window rather than simple maximization

**Table 6:** Comparison between the present work and selected transparent silica aerogel studies.

Study	Material/Process Feature	Reported Optical Metric	Comparison with This Work
This work	TEOS/EtOH/H_2_O/NH_4_OH sol–gel route; aging, solvent exchange and supercritical drying	C7: T_550_ = 84.9%	Baseline for comparison; focus is concentration-controlled nanostructure optimization
An et al. [17]	TEOS-derived transparent silica aerogel with hydrolysis control, silylation modification, and ambient-pressure drying	Broad-spectrum transparency of ~70% from 400 to 800 nm	Lower than C7; emphasizes scalable ambient-pressure processing and solar-window use
Marszewski et al. [18]	Nanoparticle colloidal suspension route on omniphobic liquid substrates under near-ambient conditions	Visible transmittance > 75%	Lower apparent transparency, but sample thickness and near-ambient processing route differ
Abebe et al. [19]	Hybrid silica aerogels from bridged silicon alkoxides by sol–gel processing and supercritical CO_2_ drying	Visible-region transparency > 80%; BT2 about 80% at 550 nm	Comparable but slightly lower; optimized also for ultralow thermal conductivity
Zhang et al. [23]	Catalyst selection and TMOS refinement in a one-step route	Solar-weighted transmittance of 83%	Comparable to C7, but solar-weighted transmittance is not identical to T_550_
Shi et al. [24]	Silicon-source and catalyst optimization for highly transparent SiO_2_ aerogels	70% at 550 nm for 0.25 g·cm^−3^ sample; 97.78% at 800 nm	Lower at 550 nm; higher 800 nm value is expected because scattering decreases at longer wavelengths
Xia et al. [25]	Batch addition of TMOS to tailor microstructure at similar composition and density	Transparency ratio increased from 71% to 88% at 550 nm	Slightly higher than C7; achieved by a different precursor-feeding strategy that more directly controls cluster growth

## Data Availability

The data presented in this study are contained within the article. Additional information and underlying data are available from the corresponding author upon reasonable request.

## Abbreviations

Abbreviation	Definition
BET	Brunauer–Emmett–Teller
EDS	Energy-dispersive spectroscopy
EtOH	Ethanol
FTIR	Fourier-transform infrared spectroscopy
SEM	Scanning electron microscopy
TEOS	Tetraethyl orthosilicate
T_550_	Optical transmittance at 550 nm
